# Event and Comment

**Published:** 1908-08-15

**Authors:** 


### DENTAL REGISTER.

**Vol. LXII. August 15, 1908. No. 8.**

### EVENT AND COMMENT.

### National Dental Association.

The annual meeting of this organization was held at Boston July 27th to 31st. It was well attended and a most interesting and profitable program was presented. Two half days were given to clinics, of which there were a great many more than any one could see, although the facilities furnished by the Tuft's College Dental School were much better than are usually found for such meetings. Almost every feature of practice was presented and many operations were duplicated. It is difficult to say whether such clinics are appropriate at a national gathering, where it would seem the time might be better occupied with matters of more general professional interest, or even of purely scientific or literary character. It has however been demonstrated many times that the attendance at meetings where no clinics are advertised has invariably been small. And so the management concludes that a national meeting needs the interest which the clinics give to secure a good attendance. The literary part of the program was of good quality, but seemed not to have excited any considerable enthusiasm. This can perhaps in large measure be accounted for by the fact that the program was not printed in sequential or definite order. There was one paper read that had five men on the program to discuss it, and every one of the five presented well digested written discussions; but this was the only paper having this admirable distinction, two of the papers read had not a single man on the program present in person or was represented by a written discussion. These things of course could have not been prevented by the best of managers. It would have been of great help had the essayists and attendants had some knowledge beforehand of what the papers were and where they would be read, this would have secured the attendance of such as were interested in particular papers and would undoubtedly encouraged extempore discussion. It is a very difficult matter to construct a program of men living at such great distances and expect it to be presented in the best form. There was much discussion in the hotel corridors as to the advisability of reorganizing the Association in some way to secure a larger attendance and better program, but no steps were taken to affect a different organization, and it may be that none is practicable. It is possible that an early and closer attention to the program might provide for more general attendance and interest. There were about 700 present, but most people think we need an attendance of from three to five thousand. But the immense distances and lack of program definiteness have not encouraged attendance, or the advancement of the highest and best professional interests. The profession of Boston and New England did all they possibly could in entertainment, and socially the meeting may well be considered a great success. Dr. Turner of South Carolina was elected President, and the meeting next year will be held at Birmingham, Alabama, and will be deferred to some time the last of September or the first week in October.

